# Identification of WT161 as a Potent Agent for the Treatment of Colitis by Targeting the Nucleotide-Binding Domain-Like Receptor Family Pyrin Domain Containing 3 Inflammasome

**DOI:** 10.3389/fphar.2022.780179

**Published:** 2022-03-07

**Authors:** Xiuyan Long, Xiaoyu Yu, Pan Gong, Xiaoyan Wang, Li Tian

**Affiliations:** Department of Gastroenterology, The Third Xiangya Hospital, Central South University, Changsha, China

**Keywords:** WT161, NLRP3 inflammasome, inflammatory bowel disease, DSS-induced, HDAC6

## Abstract

Inflammatory bowel diseases (IBD), including Crohn’s disease and ulcerative colitis (UC), are chronic and recurrent intestinal inflammatory disorders. Numerous studies have revealed that the nucleotide-binding domain-like receptor family pyrin domain containing 3 (NLRP3) inflammasome plays a pivotal role in the pathogenesis of IBD, and inhibition of the NLRP3 inflammasome alleviates colitis in experimental animals. Our previous study showed that C646, an inhibitor of histone acetyltransferase p300, has a protective role in dextran sulfate sodium (DSS)-induced colitis by targeting the NLRP3 inflammasome, making us further study the inhibitors of histone deacetylases (HDACs) in the treatment of colitis. In this study, we have shown that WT161, an inhibitor of HDAC6, exerts a protective role in a colitis model, blocks NLRP3 inflammasome activation, disrupts ASC speck formation, and decreases the expression of NLRP3. This study uncovers a new inhibitor of the NLRP3 inflammasome and suggests its potential application in the treatment of active IBD.

## Introduction

Inflammatory bowel diseases (IBD), including Crohn’s disease and ulcerative colitis (UC), are chronic and recurrent intestinal inflammatory disorders (Hodson, 2016). Although the precise pathogenesis of IBD remains unclear, it is now widely suggested that genetic-environment-mediated dysregulation of the mucosal immune response contributes to its occurrence. The morbidity and burden of IBD are increasing worldwide (Kaplan, 2015; Kaplan and Ng, 2016). The limitations of drugs currently used for IBD treatment, such as aminosalicylic acid, corticosteroids, biologicals, and immunosuppressive agents (Lamb et al., 2019), have prompted researchers to develop new drugs that are suitable for long-term use with fewer side effects. Small-molecule inhibitors have attracted increasing interest as potential agents for the treatment of IBD.

Numerous studies have revealed that the nucleotide-binding domain-like receptor family pyrin domain containing 3 (NLRP3) inflammasome plays a pivotal role in the pathogenesis of IBD (Kanneganti, 2017; Mao et al., 2018). The NLRP3 inflammasome is a cytosolic protein complex that consists of NLRP3, apoptosis-associated speck-like protein containing a CARD (ASC) and caspase-1 (Agostini et al., 2004). Once activated, NLRP3 recruits ASC and caspase-1 to form a large complex, leading to the conversion of precursors prointerleukin-1β and prointerleukin-18 into active interleukin (IL)-1β and IL-18, respectively, as well as inducing cell death. Researchers have indicated that single-nucleotide polymorphisms in the *NLRP3* gene are associated with susceptibility to UC (Hanaei et al., 2018; Zhou et al., 2021), and NLRP3 inflammasome activity is increased in some patients with UC and Crohn’s disease (McAlindon et al., 1998; Coccia et al., 2012). Moreover, selective blockade of the NLRP3 inflammasome could ameliorate colonic inflammation in a colitis model (Chen et al., 2021; Song et al., 2021). Thus, the NLRP3 inflammasome may be a promising target for future treatment of IBD.

Pharmacological inhibition of the NLRP3 inflammasome plays a protective role in colitis (Bauer et al., 2010). In our previous study, we found that the histone acetyltransferase inhibitor C646 inhibits NLRP3 inflammasome activity and exerts anti-inflammatory effects in dextran sulfate sodium (DSS)-induced colitis mice by targeting the NLRP3 inflammasome (Xu et al., 2021). Furthermore, Magupalli et al. (2020) found that histone deacetylase 6 (HDAC6) promotes the activation of the NLRP3 inflammasome (Magupalli et al., 2020), which draws our focus to HDAC6 inhibitors. Based on these studies, we suspect that the HDAC6 inhibitor WT161 can inhibit the activation of NLRP3 inflammasome and alleviate colitis in DSS-treated mice.

In this study, we found that WT161 exerts a protective role in a colitis model, blocks NLRP3 inflammasome activation, disrupts ASC speck formation, and decreases NLRP3 expression. This study uncovers a new inhibitor of the NLRP3 inflammasome and suggests its potential application in the treatment of active IBD.

## Materials and Methods

### Reagents and Antibodies

WT161 (cat. no. 1206731-57-8) from Selleck Chemicals; DSS (cat. no. 0216011080, 36,000–50,000 molecular weight) from MP Biomedicals; ultrapure lipopolysaccharide (LPS) (*E. coli* 0111: B4, cat. no. tlrl-3pelps), ATP (cat. no. tlrl-atpl), and nigericin (cat. no. tlrl-nig), monosodium urate (MSU) (cat. no. tlrl-msu) from InvivoGen; Protein A/G PLUS-Agarose (cat. no. sc-2003) from Santa Cruz; mouse immunoglobin IgG protein (cat. no. ab198772) from Abcam, and cell lysis buffer (CLB) (cat. no. 9803) from Cell Signaling Technology; mouse IL-1β (cat. no. 88–7013), IL-6 (cat. no. 88-7064-88) ELISA kits (Thermo Fisher).

Anti–IL-1β (1:1,000, AF-401-NA; RRID: AB_416,684) was obtained from RD System; anti–caspase-1 (1:1,000, ab179515) was from Abcam; anti-NLRP3 (1:1,000, Cryo-2) and ASC (1:1,000, AL177) were purchased from Adipogen; anti–β-actin (1:10,000, BH10D10), anti–p-p65 antibody (1:1,000, 3033), and anti-p65 antibody (1:1,000, 8242) were purchased from Cell Signaling Technology; DyLight 488–labeled secondary antibody (1:50, A120-100D2) from InvivoGen.

### Mice

All procedures were performed in accordance with the Institutional Animal Care and Use Committee of the Central South University. Male C57BL/6 mice (6–8 weeks old) were purchased from Hunan SJA Laboratory 100 Animal Co. Ltd (Changsha, China), and all animal experiments were carried out in the Department of Laboratory Animals of Central South University under SPF conditions.

### DSS-Induced Colitis Model and Treatment

The acute colitis model induced by DSS is widely used in studies of IBD (Perše and Cerar, 2012), and the clinical and histopathological features of acute DSS-induced colitis reflect those seen in human active IBD, such as weight loss, diarrhea, hematochezia, cryptitis, and crypt abscesses. Male C57BL/6J mice (6 weeks old) were randomly divided into four groups after acclimation for a week: control group (DMSO + H_2_O, *n* = 5), DSS group (DMSO + DSS, *n* = 12), low dose of WT161 treated DSS group (WT161-10 mg/kg + DSS, *n* = 12), and high dose of WT161 treated DSS group (WT161-20 mg/kg + DSS, *n* = 12). The mice were fed with 3% DSS or H_2_O in drinking water (Yoshihara et al., 2006; Alex et al., 2009; Shan et al., 2021) for up to 10 days (day 1–day 10). Mice in WT161 treated groups received daily intraperitoneal injections of 10 or 20 mg/kg WT161 dissolved in dimethyl sulfoxide (DMSO) from day 1 to day 10, while the control and DSS groups were intraperitoneally injected with the same solvents daily. To assess the disease activity index (DAI) score, body weight and stool were recorded daily until sacrifice. It is evaluated based on the following score system (Alex et al., 2009): *1*) weight loss (compared with day 1): 0 (≤1.0%), 1 (1.0–5.0%), 2 (5.0–10.0%), 3 (10.0–15.0%), and 4 (>15.0%); 2) stool consistency or diarrhea: 0 (normal), 1 (soft stools), 2 (loose stools), 3 (stools attached to the anus), and 4 (watery diarrhea); 3) blood in stool: 0 (negative), 1 (weak positive), 2 (positive), 3 (strong positive), and 4 (gross blood in stool). A urine fecal occult blood test box was used to detect blood in the stool. On day 10, the mice were sacrificed, and the colon was collected for colon length measurement, colon explant culture, and histological analysis.

### Histological Analysis

The 0.3–0.5 cm distal colonic segment close to the rectum was fixed in 4% paraformaldehyde for 24 h for histological analysis. The slides were prepared and stained with H&E and examined under a Nikon ECL IPSE Ci biological microscope. The images were collected using a Nikon DS-U3 digital camera. Histological scores were evaluated according to a previously published study (Obermeier et al., 1999). Epithelium (E): 0 (normal), 1 (loss of goblet cells), 2 (loss of goblet cells in large areas), 3 (loss of crypts), 4 (loss of crypts in large areas); infiltration (I): 0 (no infiltrate), 1 (infiltrate around crypt basis), 2 (infiltrate reaching to L. muscularis mucosae), 3 (extensive infiltration reaching the L. muscularis mucosae, and thickening of the mucosa with abundant edema), and 4 (infiltration of the L. submucosa). The histological scores were the sum of the epithelium and infiltration scores.

### Colon Explant Culture

The next 0.3–0.5 cm distal colonic segment (with the same weight) close to the rectum was washed with saline briefly and then washed three times with cold RPMI-1640 (Gibco) containing penicillin G (200 μg/ml) and streptomycin (200 μg/ml) to remove the residual bacteria. They were then cultured in RPMI-1640 supplemented with 10% FBS, penicillin G (200 μg/ml), and streptomycin (200 μg/ml) at 37°C with 5% CO_2_ for 24 h. Pro-inflammatory cytokines in the supernatant were detected by ELISA.

### Cell Culture

Peritoneal macrophages from healthy SPF C57BL/6J mice (6–8 weeks old) were elicited by i.p. injection of 3 ml 3% thioglycolate broth. Macrophages were harvested 3 days later by i.p. instillation and retraction of peritoneal lavage with 10 ml RPMI-1640 medium. Cells were purified by adherence and cultured in RPMI-1640 medium supplemented with 10% FBS, 100 U/ml penicillin, and 100 μg/ml streptomycin at 37°C in a humidified incubator with 5% CO_2_.

### LPS + DSS Stimulation

Peritoneal macrophages were seeded overnight in 24-well (3–4 × 10^5/well) or six-well plates (2 × 10^6/well). After 24 h, mouse peritoneal macrophages were stimulated with LPS (100 ng/ml) for 3 h, followed by treatment with DMSO or WT161 (5 μM, dissolved in DMSO) for 30 min. Next, the cells were treated with 3% DSS for 24 h. IL-1β and IL-6 in the supernatant were detected using ELISA kits.

### NLRP3 Inflammasome Activation and Analysis

Peritoneal macrophages were seeded overnight in 24-well (3–4 × 10^5/well) or six-well plates (2 × 10^6/well). Mouse peritoneal macrophages were primed with LPS (100 ng/ml) for 3 h, followed by treatment with DMSO or WT161 (0, 1, 3, or 5 μM, dissolved in DMSO) for 30 min, and then stimulated with 5 mM ATP (1 h), 10 μM nigericin (1 h), or 200 μg/ml MSU (6–8 h). To evaluate NLRP3 activation, the release of IL-1β detected by ELISA or the expression of cleaved caspase-1 p10 and cleaved IL-1β p17 detected by western blot were analyzed in the supernatant.

### Western Blot

For cell proteins, cells were lysed with CLB supplemented with a protease inhibitor cocktail and phenylmethylsulfonylfluoride (PMSF), and then the cell lysates were centrifuged at 12,000 g for 5 min at 4°C. For tissue protein analysis, the mouse colon and sodium dodecyl sulphate (SDS) lysate were lysed at a ratio of 10 mg:200 μl followed by ultrasound and grinding, centrifuged at 12,000 g for 10 min, and the supernatant was aspirated. After protein quantitative analysis, the extracts were boiled and dissolved in SDS loading buffer for western blot. Equal amounts of extracts (all proteins in the supernatant of each well, 10 μg protein extracted from cell lysate or colonic tissue) were separated by 10% SDS-PAGE, and then transferred onto 22-mm PVDF membranes (Merck Millipore, ISEQ00010) for immunoblot analysis. The signal intensity was detected using the ImageJ software. The data were standardized to β-actin expression levels.

### Quantitative PCR

Peritoneal macrophages were seeded overnight in 24-well plates (3–4 × 10^5/well). The next day, the cells were treated with or without WT161 (5 μM) for 30 min, followed by LPS (100 ng/ml) for 0, 2, 4, or 8 h. After stimulation, total mRNA was extracted using the Fast 2000 kit (FASTAGEN). Then, RNA was reverse-transcribed to complementary DNA using TransScript All-in-One First-Strand cDNA Synthesis SuperMix for RtPCR kit (TransGen) for quantitative PCR analysis. Quantitative PCR was performed using SYBR Green (Vazyme Biotech) on a LightCycler 480 (Roche Diagnostics). The data were standardized to β-actin expression levels. The relative expression changes were calculated using the 2−ΔΔCT method. Specific primers for each gene were as follows:

*NLRP3* forward: 5′-TGG​ATG​GGT​TTG​CTG​GGA​T-3′,

reverse: 5′-CTG​CGT​GTA​GCG​ACT​GTT​GAG-3′;

*Caspasse-1* forward: 5′-ACAAGGCACGGG ACCTATG-3′,

reverse: 5′-TCC​CAG​TCA​GTC​CTG​GAA​ATG-3′;

*ASC* forward: 5′-CTT​GTC​AGG​GGA​TGA​ACT​CAA​AAT​T-3′,

reverse: 5′-GCCATACGACTCCAG ATAGTAGC-3′;

*β-actin* forward: 5′-AGT​GTG​ACG​TTG​ACA​TCC​GT-3′,

reverse: 5′-GCA​GCT​CAG​TAA​CAG​TCC​GC-3′.

#### ASC Oligomerization

Peritoneal macrophages were seeded overnight in six-well plates (2 × 10^6/well). The next day, the cells were primed with LPS (100 ng/ml) for 3 h, followed by DMSO or WT161 (5 μM, dissolved in DMSO) for 30 min, and then stimulated with 10 μM nigericin for 1 h. After stimulation, cells were rinsed in PBS and 100 μl ice-cold Triton buffer (50 mM Tris-HCl (pH 7.5), 150 mM NaCl, 0.5% Triton X-100) with 0.1 mM phenylmethylsulfonylfluoride (PMSF). The cells were centrifuged at 6,000 g for 15 min at 4°C. The supernatant was resuspended in SDS loading buffer as a soluble sample. Insoluble samples (pellets) were washed twice with Triton buffer and resuspended in 200 μl Triton buffer containing 0.1 mM PMSF. Disuccinimidyl suberate (2 mM) was added to the resuspended pellets and incubated at 37°C for 30 min. The samples were then re-centrifuged at 6,000 g for 15 min at 4°C and suspended in SDS loading buffer. Both samples were then collected for western blot. The extracts (10 μg) from each sample were separated by 10% SDS-PAGE.

### ASC Speck Formation

The peritoneal macrophages were cultured overnight on chamber slides. The following day, the cells were primed with LPS (100 ng/ml) for 3 h, followed by treatment with DMSO or WT161 (5 μM, dissolved in DMSO) for 3 h, then stimulated with 10 μM nigericin or 5 mM ATP for 1 h. Cells were then fixed with 4% paraformaldehyde for 10 min, and permeabilized with 0.1% Triton X-100. After blocking with 5% BSA, cells were incubated with anti-ASC antibody (1:200) overnight at 4°C in PBS containing 5% BSA and DyLight 488-labeled secondary antibody (1:50) for 1 h in PBS containing 5% BSA. After thorough washes, the slides were mounted with mounting medium containing DAPI. Analyses were carried out using a fluorescence microscope (Nikon Ti2-U).

### Statistical Analysis

All results are expressed as mean ± SD. Statistical analysis was performed using one-way ANOVA with Bonferroni’s test or two-way ANOVA with Bonferroni’s multiple comparisons test for the groups test in GraphPad Prism software 8.0. Statistical significance was defined as *p* < 0.05, with increasing levels of confidence displayed as *p < 0.05, **p < 0.01, ***p < 0.001, and ****p < 0.0001.

## Results

### WT161 Alleviated Intestinal Inflammation in a DSS-Induced Colitis Model

**FIGURE 1:**
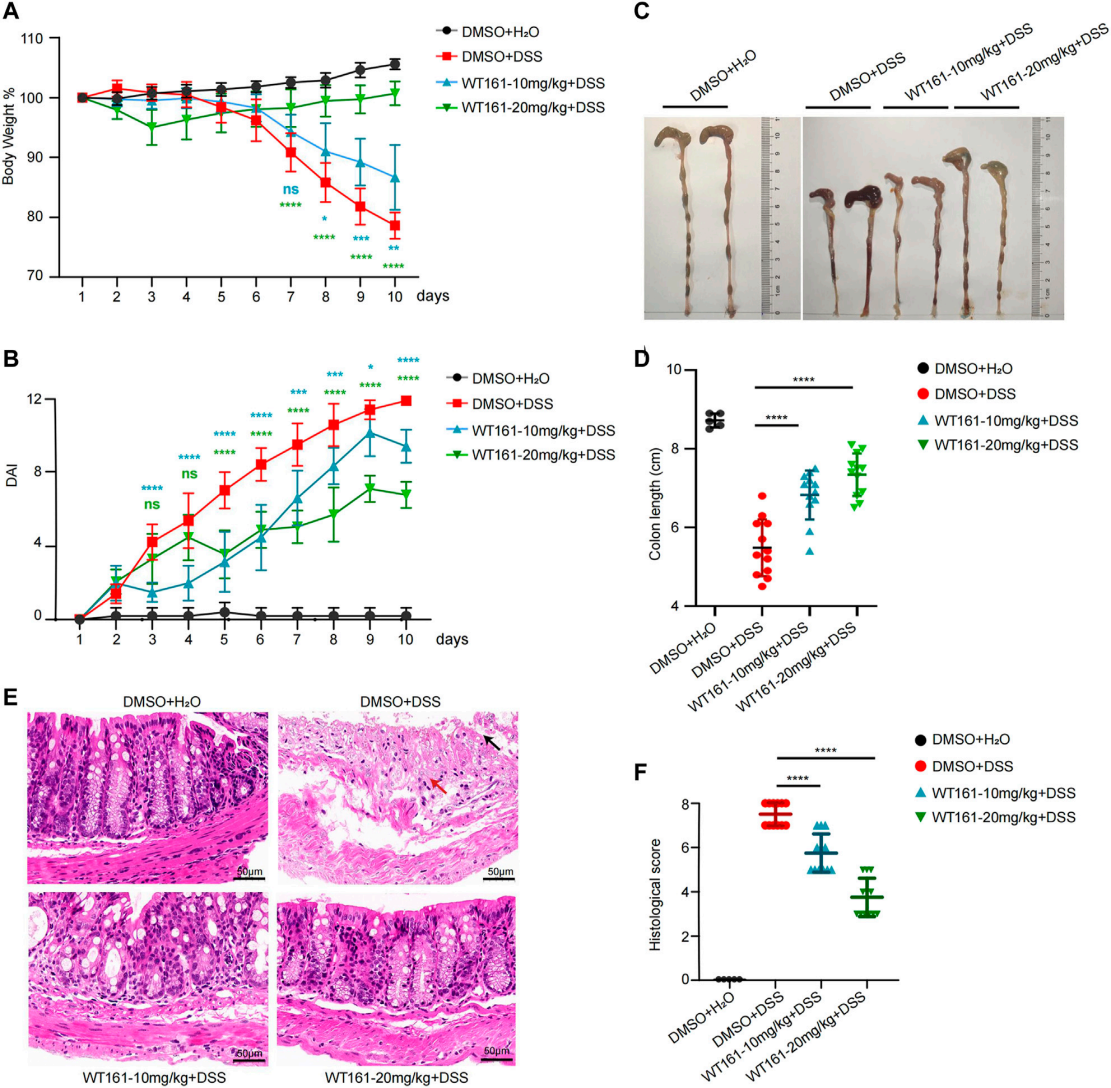
WT161 alleviated intestinal inflammation in DSS-induced colitis model. **(A–F)** The colitis model was induced in C57BL/6J WT mice by administrating 3% DSS in drinking water from day 1 to day 10. WT161 (10 or 20 mg/kg) or dimethyl sulfoxide (DMSO) were given by intraperitoneal injection from day 1 to day 10 (DMSO + H_2_O group, *n* = 5; other groups, *n* = 12). Weight change **(A)** and disease activity index (DAI) **(B)** were monitored every day. Gross morphology imaging of the colons **(C)**, measurement of the colon lengths **(D)**, representative imaging of H&E-stained colons **(E)**, and histological analysis **(F)** of colitis were performed at the end of animal experiments. Damaged crypt structure (red arrow), destroyed epithelial layer (black arrow). Scale bars: 50 μm (magnification ×300). The image and data shown in **(A–F)** are representative of three independent experiments. For **(A,B)**, the *p*-values were calculated using two-way ANOVA with the Bonferroni’s multiple comparisons test (compared with the DMSO + DSS group); for **(D,F)**, the *p*-values were calculated using one-way ANOVA with the Bonferroni’s test (compared with the DMSO + DSS group). *p < 0.05; **p < 0.01; ***p < 0.001; ****p < 0.0001; while *p* > 0.05 displayed as ns. DSS, dextran sulfate sodium; DMSO, dimethyl sulfoxide.

To investigate the function of WT161 in the treatment of NLRP3-related diseases, DSS-induced colitis was used. Based on previously reported pharmacokinetic properties, WT161 was formulated for i.p. administration once a day at a dose of 10 or 20 mg/kg in 3% DSS-treated mice from day 1 to day 10. Body weight, grossly bloody stools, and stool consistency were recorded daily. Compared to that of the control group, the WT161 treatment group showed improved weight loss (Figure 1A). Accordingly, WT161 reduced the disease activity index (DAI) score, which included weight loss, diarrhea, and gross bleeding in a dose-dependent manner (Figure 1B). Colon length, which reflects the severity of colitis, was longer after WT161 treatment (Figures 1C,D). Consistently, histopathological analysis (hematoxylin and eosin [H&E] staining) showed that WT161 administration decreased the loss of goblet cells and alleviated disruption of crypt structure and mucosal epithelium (Figure 1E), which was also confirmed by the lower histological injury score (Figure 1F). Taken together, these data indicate that WT161 exerts a protective role in a DSS-induced colitis animal model.

### WT161 Decreased the Levels of Pro-Inflammatory Factors in the Colonic Tissue of DSS-Induced Colitis Mice and Activated Peritoneal Macrophages Induced by LPS + DSS

**FIGURE 2:**
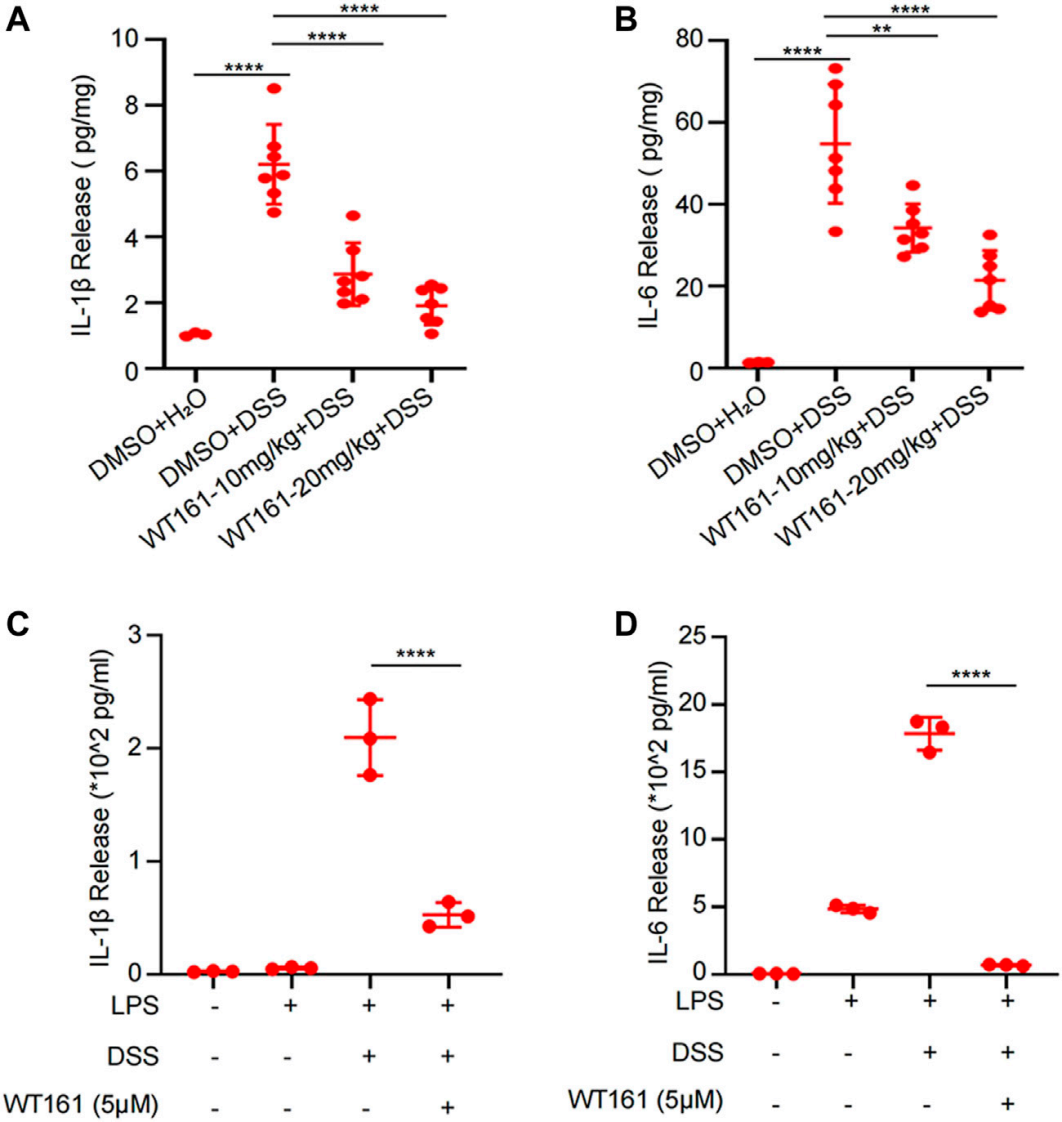
WT161 decreased the levels of the pro-inflammatory factors in the colonic tissue of DSS-induced colitis mice and activated peritoneal macrophages induced by LPS + DSS. **(A,B)** The colonic explants isolated from mice in the animal experiments were cultured for 24 h, and the interleukin (IL)-1β **(A)** and IL-6 **(B)** levels in the supernatant was detected by ELISA. For comparison, we extracted the same weight of colon explants (DMSO + H_2_O group, *n* = 3; other groups, *n* = 7). **(C,D)** The IL-1β **(C)** and IL-6 **(D)** levels in the supernatant culture of LPS + DSS-activated peritoneal macrophages. For **(A–D)**, the *p*-values were calculated using one-way ANOVA with the Bonferroni’s test. **p < 0.01; ****p < 0.0001. DSS, dextran sulfate sodium; DMSO, dimethyl sulfoxide.

Aberrant and excessive activation of some cytokines is involved in the pathogenesis of IBD (Neurath, 2014), among which IL-1β and IL-6 have been regarded to play a prominent role in the initiation and maintenance of colonic inflammation, respectively (Neurath, 2014). We then evaluated the effect of WT161 on IL-1β and IL-6 in the supernatant culture of colonic explants isolated from DSS-induced colitis mice and activated peritoneal macrophages induced by LPS + DSS. The levels of IL-1β and IL-6 in the supernatant were detected by ELISA after 24 h of colonic tissue culture. WT161 decreased the release of IL-1β (Figure 2A) and IL-6 (Figure 2B) in the colonic explants, and similar results were found in LPS + DSS-activated peritoneal macrophages (Figures 2C,D). Taken together, WT161 disturbed the release of IL-1β and IL-6, thereby inhibiting inflammation.

### WT161 Decreased NLRP3 Inflammasome Activation in Peritoneal Macrophages

**FIGURE 3:**
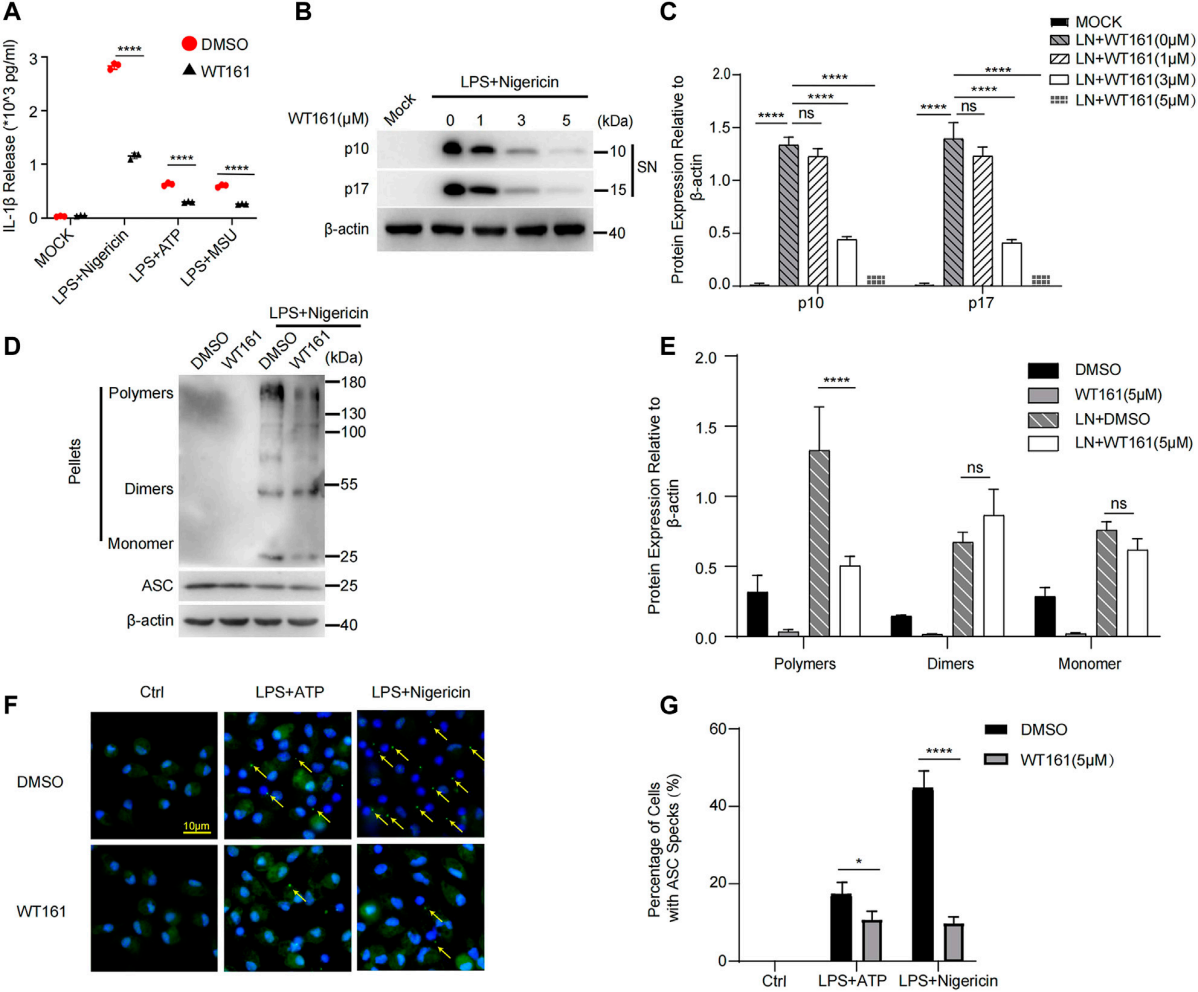
WT161 decreased NLRP3 inflammasome activation in the peritoneal macrophages. **(A)** Mouse peritoneal macrophages were pre-treated with WT161, and then primed with lipopolysaccharide (LPS), followed by stimulation with nigericin. The interleukin (IL)-1β in the supernatant was detected by ELISA. **(B,C)** The level of cleaved caspase-1 (p10) and mature IL-1β (p17) upon nigericin stimulation in the supernatant (SN) by WT161 treatment was analyzed by western blot **(B)** and quantitative analysis **(C)**. **(D)** Immunoblot analysis of ASC oligomerization in lysates of LPS-primed peritoneal macrophages treated with WT161 and then stimulated with nigericin. **(E)** Quantitative analysis of ASC oligomerization. **(F)** Immunofluorescence microscopy analysis of ASC Speck (arrow) in LPS-primed peritoneal macrophages treated with or without WT161, and then stimulated with ATP or nigericin. Scale bars: 10 μm. **(G)** Percentage of cells with ASC specks. The image and data shown in **(A–F)** are representative of three independent experiments. For **(A,C,E,G)**, the *p*-values were calculated using two-way ANOVA with the Bonferroni’s multiple comparisons test. *p < 0.05; ****p < 0.0001; while *p* > 0.05 displayed as ns. DMSO, dimethyl sulfoxide; LN, LPS + nigericin; SN, supernatant; MOCK, blank control.

Considering that IL-1β mainly depends on the activation of the NLRP3 inflammasome (Mao et al., 2018) and the fact that HDAC6, the target of WT161, is indispensable for the activation of inflammasomes (Magupalli et al., 2020), we investigated the effect of WT161 on NLRP3 inflammasome. The NLRP3 inflammasome can be activated by a wide variety of unrelated stimuli such as nigericin. ATP and MSU are other commonly used activators of the NLRP3 inflammasome by acting on the plasma membrane P2X7 purinergic receptor to promote Ca^2+^ and Na^+^ influx or by causing lysosomal rupture, respectively. WT161 reduced the release of IL-1β upon nigericin, ATP, and MSU stimulation (Figure 3A). We observed a reduction in cleaved caspase-1 (p10) and mature IL-1β (p17) (Figures 3B,C) upon nigericin stimulation in the supernatant by WT161 treatment in a dose-dependent manner.

Upon treatment with NLRP3 activators, NLRP3 undergoes initial self-oligomerization, after which NLRP3 recruits ASC, leading to ASC oligomerization, which is critical for subsequent caspase-1 activation. Thus, we tested ASC oligomerization to further evaluate the effect of WT161 on the activation of the NLRP3 inflammasome. Using disuccinimidyl suberate crosslinking, we found that WT161 effectively inhibited the formation of ASC polymers (Figures 3D,E). This result was confirmed by immunofluorescence (Figures 3F,G).

### WT161 Inhibited the Expression of NLRP3 and Negatively Modulated NF-κB Signaling

**FIGURE 4:**
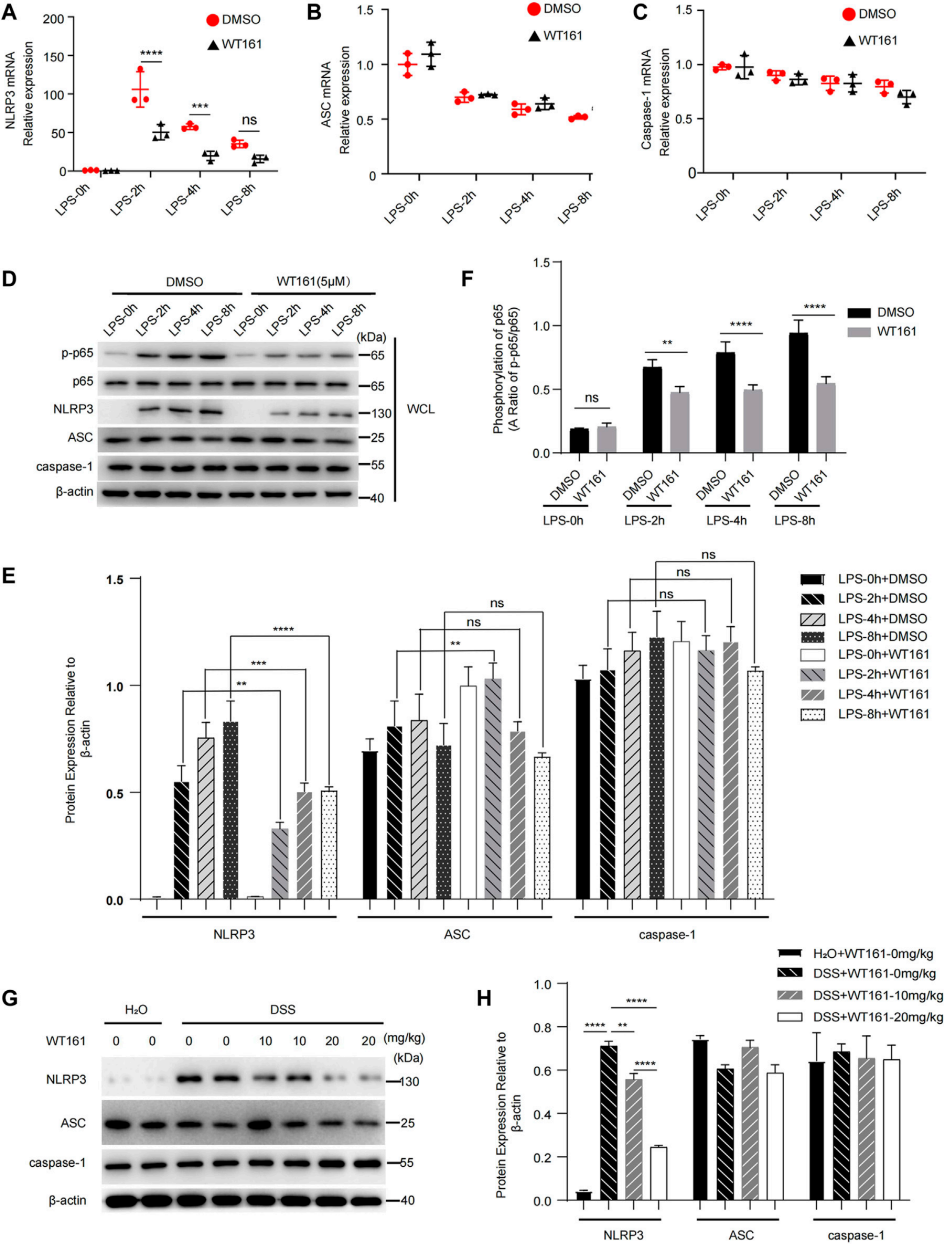
WT161 inhibited the expression of NLRP3 and negatively modulated NF-κB signaling. **(A–C)** Relative mRNA expression of NLRP3 **(A)**, ASC **(B)**, and caspase-1 **(C)** in the primary peritoneal macrophages treated with WT161 (5 μM) for 30 min and primed with lipopolysaccharide (LPS) for the indicated hours. **(D)** Immunoblot analysis of p-p65, p65, NLRP3, ASC, and caspase-1 in the mouse peritoneal macrophages with same treatment in **(A–C)**. **(E)** Relative expression to β-actin of NLRP3, ASC, caspase-1 in the primary peritoneal macrophages. **(F)** Ratio of p-p65/p65 in the primary peritoneal macrophages. **(G)** Immunoblot analysis of NLRP3, ASC, and caspase-1 in the colonic tissue isolated from DSS-induced colitis mice. **(H)** Relative expression to β-actin of NLRP3, ASC, and caspase-1 in the colonic tissue isolated from DSS-induced colitis mice. The image and data shown in **(A** **–H)** are representative of three independent experiments. For **(A–** **C,** **E,** **F,** **H)**, the *p*-values were calculated using two-way ANOVA with the Bonferroni’s multiple comparisons test. **p < 0.01; ***p < 0.001; ****p < 0.0001; while *p* > 0.05 displayed as ns. DMSO, dimethyl sulfoxide; WCL, whole cell lysate.

Finally, we aimed to explore the effect of WT161 on the expression of NLRP3, ASC, and caspase-1. The priming process of NLRP3 activation is induced by various pathogen-associated molecular patterns or cytokines, leading to activation of NF-κB signaling, which induces the expression of IL-1β and NLRP3 (Bauernfeind et al., 2009). We treated macrophages with WT161 before LPS stimulation and analyzed the mRNA expression of NLRP3, ASC, and caspase-1. Intriguingly, the qPCR results showed that WT161 significantly reduced the mRNA level of NLRP3, with no effects on ASC and caspase-1 (Figures 4A–C). Consistently, western blot analysis showed that WT161 only decreased the NLRP3 protein expression, without affecting ASC and caspase-1 expression in the LPS-treated peritoneal macrophages (Figures 4D,E). Moreover, WT161 reduced the LPS-induced increase in p-p65 and p-p65/p65 (Figures 4D,F), a direct indicator of activated NF-κB signaling. To further demonstrate this effect, we collected the protein extracted from colonic tissue isolated from the DSS-treated mice and detected the expression of these proteins. We observed that DSS induced the expression of NLRP3 in the colon tissues, whereas the WT161-treated group had a lower expression of NLRP3 (Figures 4G,H). However, ASC and caspase-1 were not affected by WT161 (Figures 4G,H). Taken together, these results imply that WT161 inhibits the expression of NLRP3 and negatively modulates NF-κB signaling during the priming process.

## Discussion

In this study, we found that the HDAC6 inhibitor WT161 exerts a protective effect in a DSS-induced colitis murine model and blocks NLRP3 inflammasome activation, as IL-1β release and ASC oligomerization were inhibited in the WT161-treated group. WT161 decreased the NLRP3 expression by inhibiting the NF-κB signaling. Thus, our study suggests that WT161 could be a promising agent for the treatment of active IBD.

WT161 has been regarded as a remarkable anti-tumor agent for several types of cancers, including multiple myeloma (Hideshima et al., 2016; McClure et al., 2018; García-Guerrero et al., 2021), breast cancer (Hideshima et al., 2017), osteosarcoma (Sun et al., 2021), and retinoblastoma (Sun et al., 2019) by affecting the regulation of CD38, growth factor receptors, PTEN, or X-linked inhibitor of apoptosis protein (XIAP) in tumor cells. However, the role of WT161 in the pathology of IBD or inflammatory diseases has not yet been explored. In this study, we found that WT161 ameliorated DSS-induced colitis by targeting the NLRP3 inflammasome, expanding its potential application in inflammatory diseases.

Network of cytokines including IL-1β, TNF-α, IL-6, IL-10, IFN-γ, and IL-18, have been reported to regulate mucosal inflammation in colitis, and IL-6, TNF-α are viewed as therapeutic targets in IBD (Neurath, 2014, 2017; Giraldez et al., 2021). In this study, we focused on the role of WT161 on the NLRP3 inflammasome, so we detected the downstream effector of NLRP3 inflammasome, IL-1β, and the nonspecific effector, IL-6, *in vitro* and *in vivo*. As TNF-α is the most frequent studied cytokine in mucosal inflammation, we did not further detect it. We supposed that WT161 could down-regulate TNF-α expression since it could suppress NF-κB signaling.

Although the role of the NLRP3 inflammasome in murine IBD models with NLRP3, ASC, or caspase-1 deficiency remains controversial (Siegmund et al., 2001; Allen et al., 2010; Bauer et al., 2010; Dupaul-Chicoine et al., 2010; Zaki et al., 2010; Bauer et al., 2012; Mao et al., 2018), inhibitors targeting the NLRP3 inflammasome, such as MCC950, oridonin, and INF39, have anti-inflammatory effects in colitis models (Chen et al., 2021). Consistent with previous studies, in an animal colitis model, WT161 improved weight loss and DAI score induced by DSS in this study. Moreover, the WT161-treated group showed longer colon length and mitigated intestinal inflammatory injury. The improvement in efficiency was positively related to the dose of WT161. Thus, WT161 exerted protective effects in a DSS-induced murine model. The role of the NLRP3 inflammasome in murine IBD models remains controversial and has been extensively discussed (Mao et al., 2018; Chen et al., 2021). Researchers have attempted to analyze and explain this contradiction. The NLRP3 inflammasome is an important part of innate immunity that resists the invasion of pathogenic microorganisms, and defect or excessive inhibition of inflammasomes leads to more severe colitis. In addition, IL-18 promotes epithelial repair, and the defect or excessive inhibition of inflammasomes leads to lower IL-18 levels. In addition, researchers have found that the intestinal flora plays a role in the controversial data from studies of NLRP3 inflammasome and IBD. In short, the reasons for this are complex, and further studies are needed. Targeting the NLRP3 inflammasome might be an effective way to treat IBD despite contradictions (Song et al., 2021).

Although we speculate that WT161 modulates NF-κB signaling and inhibits the expression of NLRP3, resulting in the inhibition of NLRP3 inflammasome activation based on the results of this study, the function of WT161 as an inhibitor of HDAC6 should not be neglected. Acetylation participating in NLRP3 inflammasome activation (Zhao et al., 2019; Liu et al., 2020) regulates NLRP3 acetylation a good orientation to identify NLRP3 inflammasome inhibitors. HDAC regulate the protein acetylation process, together with histone acetyltransferase. Thus, these corresponding inhibitors could be effective agents for regulating NLRP3 inflammasome activation. These effects have been reported in the recent studies. The study of Junya Kaneko showed that Ky-2, a hybrid-compound HDAC inhibitor, regulates M1 macrophage polarization by downregulating the expression of NLRP3 and the IL-1β-encoding gene in THP-1 cells (Kaneko et al., 2018). IL-6 is an important pro-inflammatory cytokine secreted by M1 macrophages (Atri et al., 2018). The release of IL-6 was inhibited after WT161 treatment (Figures 2B,D). Whether WT161 influences macrophage polarization *in vivo* and *in vitro* needs to be further explored. In addition, Maartje et al. reported that butyrate, an inhibitor of class I HDAC, decreases the production of IL-1β in MSU-stimulated peritoneal macrophages by inhibiting NLRP3 activation (Cleophas et al., 2016). In this study, we found that treatment with the selective HDAC6 inhibitor WT161 effectively decreased the release of IL-1β and ASC oligomerization upon NLRP3 activation. Mechanistically, WT161 decreased NLRP3 expression and negatively modulated NF-κB signaling. Our data are consistent with a previous report that HADC6, the target HADC of WT161, promotes NLRP3 activation by mediating an aggresome-like mechanism and is indispensable for the assembly of inflammasomes (Magupalli et al., 2020). Given that HDAC6 exerts its promotive effects by mediating the microtubule transport of trans-Golgi network-localized NLRP3 (Magupalli et al., 2020), whether WT161 affects NLRP3 assembly through the same mechanism remains to be investigated. We speculate that WT161 might affect the ubiquitin-binding of HDAC6, the critical activity of HDAC6 required for NLRP3 inflammasome activation, but this hypothesis requires more data for testing.

Studies have found that HDAC1, 2, 3, 6, and 9 play important pro-inflammatory roles in the initiation and progression of IBD (Felice et al., 2015). In these studies, five HDAC6 inhibitors, potassium acetate (Lu et al., 2016), tubacin (de Zoeten et al., 2011), BML-281 (Do et al., 2017), LTB2 (Liu et al., 2017), and CKD-506 (Lee et al., 2020), could alleviate DSS-induced murine colitis by targeting microtubule disassembly, regulating the functions of Foxp3(+) T-regulatory cells, suppressing the infiltration of CD19(+) B cells into the inflamed colonic lamina propria, unknown mechanisms, or blocking NF-κB signaling, respectively. Consistent with these studies, we found that WT161 alleviates colitis by inhibiting the NLRP3 inflammasome in this study, providing a new vision for the potential application of this inhibitor.

Overall, our data suggest that WT161 alleviated colonic inflammation in DSS-induced colitis mice as an effective inhibitor of the NLRP3 inflammasome. WT161 not only decreased NLRP3 expression by suppressing NF-κB signaling but also inhibited ASC formation. In a colitis model, WT161 ameliorated intestinal injury, suggesting its potential use in the treatment of IBD. Although further mechanisms remain to be elucidated, our studies have identified WT161 as a therapeutic agent for the treatment of NLRP3-related inflammatory diseases such as IBD.

## Data Availability

The original contributions presented in the study are included in the article/Supplementary Material, further inquiries can be directed to the corresponding author.

## Abbreviations

ASC, apoptosis-associated speck-like protein containing a CARD; DAI, disease activity index; DMSO, dimethyl sulfoxide; DSS, dextran sulfate sodium; IBD, inflammatory bowel disease; IL, interleukin; NLRP3, nucleotide-binding domain-like receptor family pyrin domain containing 3; UC, ulcerative colitis.
